# Evaluating NetMHCpan performance on non-European HLA alleles not present in training data

**DOI:** 10.3389/fimmu.2023.1288105

**Published:** 2024-01-16

**Authors:** Thomas Karl Atkins, Arnav Solanki, George Vasmatzis, James Cornette, Marc Riedel

**Affiliations:** ^1^Department of Electrical and Computer Engineering, University of Minnesota, Minneapolis, MN, United States; ^2^Biomarker Discovery Group, Mayo Clinic, Center for Individualized Medicine, Rochester, MN, United States; ^3^Department of Mathematics, Iowa State University, Ames, IA, United States

**Keywords:** NetMHCpan, training bias, MHC, HLA, peptide, machine learning, neural networks

## Abstract

Bias in neural network model training datasets has been observed to decrease prediction accuracy for groups underrepresented in training data. Thus, investigating the composition of training datasets used in machine learning models with healthcare applications is vital to ensure equity. Two such machine learning models are NetMHCpan-4.1 and NetMHCIIpan-4.0, used to predict antigen binding scores to major histocompatibility complex class I and II molecules, respectively. As antigen presentation is a critical step in mounting the adaptive immune response, previous work has used these or similar predictions models in a broad array of applications, from explaining asymptomatic viral infection to cancer neoantigen prediction. However, these models have also been shown to be biased toward hydrophobic peptides, suggesting the network could also contain other sources of bias. Here, we report the composition of the networks’ training datasets are heavily biased toward European Caucasian individuals and against Asian and Pacific Islander individuals. We test the ability of NetMHCpan-4.1 and NetMHCpan-4.0 to distinguish true binders from randomly generated peptides on alleles not included in the training datasets. Unexpectedly, we fail to find evidence that the disparities in training data lead to a meaningful difference in prediction quality for alleles not present in the training data. We attempt to explain this result by mapping the HLA sequence space to determine the sequence diversity of the training dataset. Furthermore, we link the residues which have the greatest impact on NetMHCpan predictions to structural features for three alleles (HLA-A*34:01, HLA-C*04:03, HLA-DRB1*12:02).

## 1 Introduction

Antigen presentation by the major histocompatibility complex (MHC) class I and II proteins (referred to as HLA in humans) is one of the crucial steps to activating the adaptive immune response, and the genes which encode these proteins are some of the most polymorphic genes in humans (1). As a result, the epitopes presented to T cells are determined partly by the binding affinity between the peptide fragment of the antigen and the host-specific MHC protein, which is determined by the amino acid sequences of both peptide and MHC. Because of the central role of this process in adaptive immunity, the ability to predict which peptides will bind to a given MHC allele has utility in diverse fields. For example, peptide-MHC binding predictions have been used to select peptides for a cancer neoantigen vaccine and to explain asymptotic SARS-CoV-2 infection in individuals with a specific HLA-B allele (2, 3). While molecular dynamics (MD) systems exists for modelling these complexes (4, 5), the current consensus is that neural network prediction models are accurate enough at predicting binding affinity to be used in clinical settings (6). Many such tools have been developed to predict peptide binding to both MHC class I and MHC class II (6–9). Two neural-network based predictors, NetMHCpan-4.1 and NetMHCIIpan-4.0 (here on out collectively referred to as NetMHCpan) are hosted on a web server and are fast to return predictions (10). The relative popularity of NetMHCpan makes it a fitting choice for further investigation (**Supplementary Figure S1**).

Despite its popularity, NetMHCpan does not rely on any structural information about the peptide or MHC molecule, and only takes an amino acid sequences for the peptide and MHC protein as input, which limits the model’s ability to generate mechanistic explanations for its binding predictions Additionally, the tool is closed-source, exacerbating its “black box” nature and prompting investigations into potential hidden biases. A previous study has shown NetMHCpan-4.1 has a previously unreported bias toward predicting hydrophobic peptides as strong binders, suggesting the predictions of these models need to be examined closely (11).

Many times when medical and biological neural network based prediction systems have been evaluated, researchers have uncovered numerous examples of racial bias in machine learning algorithms (12–14). Furthermore, datasets from prior genomic studies often fail to capture the genetic diversity of the human population, often focusing on individuals of European descent (15–17). As these two significant effects intersect to produce models that overfit to overrepresented populations, it is vital that neural-network models be carefully investigated to determine the extent to which there is bias in the training dataset, and if it exists, the extent to which this bias affects the model predictions.

To determine the impact of training dataset bias on NetMHCpan’s predictions, we examined the geographic distribution of NetMHCpan’s training dataset and determined which populations are likely to have alleles not represented in NetMHCpan’s training dataset. We then measured the performance of NetMHCpan on alleles not present in its training dataset, and compared the performance to binding predictions for alleles present in its training dataset. To better understand these predictions, we created a map of HLA sequence space to determine the diversity of the dataset at the sequence level. Finally, for each of three MHC molecules not in NetMHCpan’s training dataset, we determined the residues of that molecule that have disproportionate impact on NetMHCpan’s predictions.

This paper presents a geographic imbalance in the HLA types present in NetMHCpan’s training data, yet fails to find a meaningful drop in the accuracy of the network’s peptide binding predictions for alleles not present in the training data compared to the accuracy of the models’ prediction on alleles present in the training dataset. Furthermore, the results suggest two possible explanations for this finding. First, while the model may be lacking in geographic diversity, the alleles represented in the training dataset cover a large range of HLA sequences. Second, the model gives attention to residues structurally involved in peptide-MHC complexes for novel alleles.

## 2 Materials and methods

### 2.1 MHC allele population demographics

Data on HLA allele population frequencies were downloaded from the National Marrow Donor Program (NMDP) (18). The dataset contains HLA-A/B/C/DRB1 population frequencies from *n* =6.59 million subjects from the United States. Population frequencies are reported 21 self-reported racial groups, which are combined into six larger ethnicity categories, given in **Supplementary Table S1**. Because NetMHCpan uses a motif deconvolution algorithm for training, there exist data points in the eluted ligand dataset where a peptide corresponds to multiple MHC alleles (10). In this case, we conservatively counted an allele as present in the training dataset if there is at least one positive example of a peptide binding to the associated cell line.

### 2.2 Evaluating NetMHCpan performance

#### 2.2.1 Evaluation datasets

In order to evaluate the performance of NetMHCpan, we used a dataset from Sarkizova et al. (19). The dataset consists of eluted ligand (EL) data for 31 HLA-A alleles, 40 HLA-B alleles, and 21 HLA-C alleles, with a median of 1,860 peptides per allele, generated by cell lines engineered to express only one HLA type. Of these alleles, 7 (A*24:07, A*34:01, A*34:02, A*36:01, C*03:02, C*04:03, and C*14:03) have no representation in NetMHCpan’s training data (binding affinity or eluted ligand). We compute but do not report the results for HLA-B, as all forty of the HLA-B alleles had some presence in the NetMHCpan training data. We have an average of 2179 peptides per MHC class I allele, with all alleles having at least 918 peptides (**Supplementary Tables S1**, **S2**).

As no similar dataset exists for MHC class II, we created an evaluation set by downloading peptides from IEDB (20). For each allele, the filters used were “Include Positive Assays”, “No T cell assays”, “No B cell assays”, and “MHC Restriction Type: [allele] protein complex.” To choose DRB1 alleles of interest, we selected alleles for which NetMHCIIpan-4.0 had eluted ligand data from a cell line engineered to express only one HLA-DRB1 allele. To obtain data for HLA-DRB1*12:02, the only HLA-DRB1 allele not in NetMHCIIpan-4.0’s training dataset for which sufficient peptide binding data exists, we use a eluted ligand dataset from cell line C1R expressing HLA-DR12/DQ7/DP4 (21). Because the cell line expressed both HLA-DRB1*12:02 and HLA-DRB3*02:02:01, Gibbs Cluster was used to separate the two groups (22) (**Supplementary Figure S2**, **Supplementary Data Sheet 6**). The group belonging to DRB1*12:02 was identified by the absence of F at P1, the absence of N at P4, and the presence of Y/F at P9. We have an average of 8094 peptides per HLA-DRB1 allele, with a minimum of 8094 (**Supplementary Table S3**).

To provide negative controls for both MHC class I and II, the real peptides were combined with randomly sampled peptides from the human proteome so that the ground truth peptides made up 1% of the final evaluation set. We found that sampling random amino acid strings compared to entire peptides made a small difference in the relative rankings of allele performance, but did not meaningfully alter our conclusions (**Supplementary Figure S3**). For the MHC class II dataset, the length distribution of the randomly generated peptides was fixed to be equal to the length distribution of the ground truth peptides.

#### 2.2.2 Log rank predictions, motif entropy correction, and AUPRC

As a result of the above preprocessing steps, we obtained a dataset for 31 HLA-A alleles, 40 HLA-B alleles, 21 HLA-C alleles, and 11 HLA-DRB1 alleles, each dataset being made up of 1% peptides experimentally verified to bind to the HLA allele in eluted ligand assays, and 99% randomly generated peptides to serve as a control (**Supplementary Figure S4**). For each allele, we used NetMHCpan-4.1 or NetMHCIIpan-4.0 to generate an eluted ligand (EL) score for each peptide in the training dataset, and ranked all peptides by their EL scores. That is, each peptide was assigned a (fractional) rank score as:


(1) $${\text{rank}}_{i}=\frac{\sum_{j=1}^{100n}(EL_{i}<EL_{j})}{100n}$$

where *EL_i_* is the EL score of the *i*-th peptide and *n* is the number of experimentally verified peptides in the dataset. Thus, peptides with higher binding scores will have lower ranks.

We then measured performance based on the distribution of log_10_ ranks for the experimentally verified peptides. For example, if the model is a perfect predictor, all real peptides will have a log_10_ rank below -2, and if the model is a random predictor, 90% of real peptides will have a log_10_ rank between 0 and -1.

To correct for any discrepancies in difficulty predicting ligands based on selectivity of the MHC binding motif, we calculated the information of the binding motif for each allele by using the Kullback–Leibler divergence, so


(2) $$I=\sum_{i=1}^{n}\sum_{a}p_{a,i}\log_{2}\left(\frac{p_{a,i}}{q_{a}}\right),$$

where *p_a,i_* is the frequency of amino acid *a* at position *i* in the allele-specific experimentally verified binding peptides, and *q_a_* is the background frequency of amino acid *a* in the human proteome (**Supplementary Figures S5–S8**). No correlation exists between the number of peptides in the allele dataset and the allele motif information, suggesting that low motif information is not a results of small sample size (**Supplementary Figure S8**).

We then performed a linear regression for the log-rank against the information (**Supplementary Figure S9**). For both MHC class I and class II, we found alleles with higher information (more predictable) motifs were associated with better predictions, as expected. Therefore, for each allele we calculated a correction factor *C* such that:


(3) $$C_{\text{allele}}=\alpha +\beta I_{\text{allele}}-\mu$$

where *I*_allele_ is the KL divergence of the allele motif against the human proteome amino acid frequency distribution, *α* and *β* are the coefficients computed from the linear regression, and *µ* is the mean of all log ranks for all alleles. Including the *µ* term ensures that our predictions remain on the same scale after subtracting the correction factor.

Additionally, because MHC proteins bind a core motif that can contain additional amino acids on the ends that do not affect the binding prediction, we encountered cases in the prediction datasets where multiple versions of a peptide contained the same core sequence. Therefore, in these cases, we chose to weight the peptides based on NetMHCpan’s reported binding core such that each core was weighted equally.

To determine a 95% confidence interval for the difference between the median of the ranks of the alleles with and without training data, a bootstrap procedure was used. Data were sampled with replacement for a number of times equal to the size of the data, and the difference between the medians of the bootstrap samples was calculated. This was repeated 10^6^ times, and the 0.025 and 0.975 quantiles were reported as the 95% confidence interval.

Finally, we calculate the AUPRC and PPV metrics for each allele. We calculate AUPRC as the area under the precision-recall curve. The precision is defined by *P* = *TP/*(*TP* + *FP*), and the recall is defined by *R* = *TP/*(*TP* + *FN*). True positives are defined as experimentally verified peptides with a motif entropy-corrected score greater than a given cutoff, and false positives as randomly generated peptides with an motif entropy-corrected score greater than a given cutoff. True negatives are defined as randomly generated peptides with a motif entropy-corrected score less than a give cutoff, and false negatives as experimentally verified peptides with a motif entropy-corrected score less than a given cutoff. We calculate the positive predictive value PPV as the number of experimentally verified peptides with corrected rank less than 0.01 divided by the number of experimentally verified peptides.

### 2.3 MDS of HLA alleles

Using the NMDP frequency database, HLA-A, B, C, and DRB1 alleles with a frequency greater than 0.01% in any population were selected (*n* = 135 HLA-A, *n* = 258 HLA-B, *n* = 66 HLA-C, *n* = 118 HLA-DRB1). The IPD-IMGT/HLA alignment tool was used to create an alignment of the selected HLA full protein sequences (23). In cases where large gaps occurred at the beginning or end of the alignment, gaps were filled with the most common amino acid occurring at that residue. Similarity between sequences was measured by summing the values of the PAM100 matrix for each pair of amino acids in the two sequences (24). Sequence distance was then measured as the difference between the maximum similarity and the computed similarity, normalized so that the maximum distance was reported. For peptides which has associated binding data, motif distance was computed as the Jenson Shannon divergence. The R cmdscale function with default parameters was used to compute the MDS (25, 26).

### 2.4 NetMHCpan residue substitution sensitivity

Here, we describe a technique similar to the occlusion sensitivity technique common in the field of computer vision. We chose the alleles HLA-A*34:01, HLA-C*04:04. and HLA-DRB1*12:02 for the following experiments, as NetMHCpan performed the poorest on these three alleles. For each allele, we used NetMHCpan to predict the eluted ligand score for all peptides found to bind to the allele in the evaluation datasets described above. Next, for residues 1-205 (29-125 for DRB1*12:02), we asked NetMHCpan to predict the eluted ligand score for all experimentally verified peptides, using a version of the MHC sequence where for each residue, each of the other 19 amino acids was substituted. From this, we took the 5 amino acids for which NetMHCpan predicted the lowest scores, and calculated the mean difference between EL scores for the mutated and unmutated predictions, as to investigate the effect of replacing residues with dissimilar amino acids. Repeating this for every residue, we then obtained a metric for the relative importance of the residue to NetMHCpan’s predictions. HLA tertiary structures were generated using PANDORA and visualized using PyMOL (4, 27).

### 2.5 Software versions

The following software versions were used: NetMHCpan (4.1), NetMHCIIpan (4.0), PANDORA (2.0), GibbsCluster (2.0), PyMol (2.6.0a0). For all tools, a local version was downloaded instead of using a web server.

## 3 Results

### 3.1 Common European Caucasian HLA types are overrepresented in NetMHCpan training data

As neural network prediction biases are often enforced by disparities in the amount of model training data, we first investigate NetMHCpan’s training dataset to determine whether the data is representative of the global population. To do this, we used allele distribution data from the National Marrow Donor Project (NMDP) (18). Codes for population groups can be found in **Supplementary Table S4**. For each population, we calculated the fraction of people who have at least one HLA-A/B/C/DRB1 allele for which there is no data in NetMHCpan’s training set.

**Figure 1:**
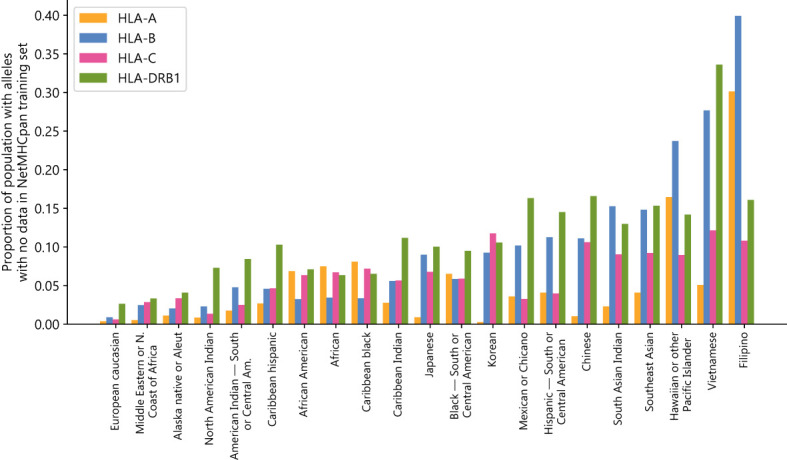
NetMHCpan training data fails to cover common HLA alleles: Proportion of populations (as defined by the National Marrow Donor Program) that have at least one HLA class A, B, C, or DRB1 allele with no data in the NetMHCpan-4.1 or NetMHCIIpan-4.0 training datasets.

There exists a substantial disparity between the most and least represented populations in NetMHCpan’s training dataset. European Caucasian individuals are most likely to see their genotypes represented in the training set, while Southeast Asian, Pacific Islander, South Asian, and East Asian individuals are least likely to have genotypes represented in the training set (**Figure 1**) (**Supplementary Data Sheet 2**). Using the NMDP categories, only 0.4%/0.9%/0.6%/2.6% of European Caucasian individuals have an HLA-A/B/C/DRB1 allele not found in NetMHCpan’s training data, while 5.1%/27.7%/12.1%/33.6% of Vietnamese individuals and 30.1%/39.3%/10.8%/16.1% of Filipino individuals have an HLA-A/B/C/DRB1 allele not found in NetMHCpan’s training data.

These disparities are not likely to have arisen by chance alone, given the fractions of the populations for which no data exists are correlated between HLA groups (**Supplementary Table S5**). For all pairs of groups there exists a positive correlation, with the strongest correlation between HLA-A and HLA-B (0.750) and the weakest correlation between HLA-A and HLA-DRB1 (0.238). Because the disparities are found in all four HLA groups examined and are correlated with each other, this suggests a common systemic factor driving the extreme imbalance of the training dataset.

### 3.2 NetMHCpan-4.1 and NetMHCIIpan-4.0 accurately predict peptide binding to novel alleles

Because there exists such a vast disparity in the representation of populations in NetMHCpan’s training data, we hypothesized NetMHCpan is overfitting to the training set, making the model unable to make accurate predictions for peptides binding to novel MHC proteins. Therefore, we investigated whether there is a decrease in prediction quality for HLA sequences not found in the training data. To do this, we performed an experiment in which NetMHCpan was tasked to predict eluted ligand binding scores for a dataset consisting of 1% peptides experimentally verified to bind to their corresponding MHC proteins and 99% randomly generated peptides, as is the standard to test MHC-peptide prediction models (10, 28). We then measured the rank of the predictions for the experimentally verified peptides, which we use as our metric for the accuracy of the predictions (after a correction for motif information described in the Methods section), as well as the area under the precision-recall curve for each set of predictions (AUPRC).

We ran the MHC class I peptide experiment on a large HLA class I eluted ligand dataset (19). In the dataset are *n* = 39617 peptides for 27 HLA-A and 18 HLA-C alleles with training data in NetMHCpan-4.1’s training set, and *n* = 8652 peptides for 4 HLA-A alleles and 3 HLA-C alleles without data in NetMHCpan-4.1’s training set. All together, these novel alleles represent up to 28.8% of HLA-A alleles, and up to 11.7% of HLA-C alleles for some populations (**Supplementary Figure S10**). Because there are no HLA-B alleles present in the dataset but absent from NetMHCpan-4.1’s training set, we do not report information about HLA-B in the results (although we do use these alleles in the information correction).

**Figure 2:**
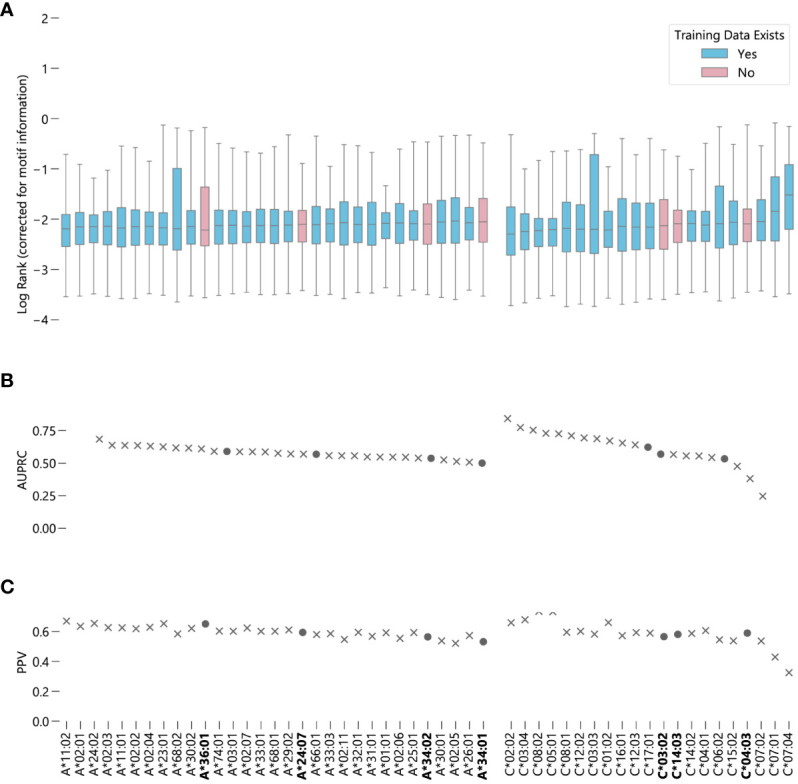
Evaluating NetMHCpan-4.1 performance on novel alleles: NetMHCpan-4.1 was tasked with separating peptides identified as true binders using LC-MS/MS (from Sarkizova et. al.) from randomly generated peptides for 52 HLA class I alleles. **(A)** Box-and-whisker plot of log ranks of the true peptides, corrected for entropy of the allele binding motif (lower is better). Whiskers show the middle 95% of data for each allele. Alleles with training data in NetMHCpan-4.1’s training dataset are shown in blue, alleles without are shown in pink. **(B)** Area under the precision-recall curve (AUPRC) for each allele. **(C)** Positive predictive value (PPV) for each allele.

NetMHCpan-4.1 accurately recalls experimentally validated peptides from a training dataset containing validated peptides and randomly generated peptides for these 7 alleles. For both HLA-A and HLA-C, the alleles for which NetMHCpan-4.1 has no training data are roughly evenly distributed amongst the other alleles in terms of performance (**Figure 2**) (**Supplementary Data Sheet 3**). Overall, the predictions of binding peptides for the alleles for which NetMHCpan-4.1 has no training data only slightly underperform compared to the predictions for alleles for which it does have data (**Supplementary Figure S11A**), with a 95% bootstrap confidence interval for the difference in the medians of the two sets being (-0.039, -0.014) (**Supplementary Figure S12A**). On average, NetMHCpan-4.1 ranks experimentally verified peptides for alleles for which data exists in its training dataset only 1.06 times higher than it ranks peptides which have no data in the training set. In almost all cases, there is a large difference between the raw EL scores between the true binders and the randomly generated peptides (**Supplementary Figure S13**). Furthermore, the general trend of the results hold without the correction for motif information (**Supplementary Figure S14**). In summary, we fail to find evidence that the imbalance in the training dataset leads a decrease in the quality of NetMHCpan-4.1 predictions for novel alleles.

In the case of MHC class II predictions, we focus exclusively on DRB1 because HLA-DR is the only MHC class II protein to vary only in the beta chain, which simplifies the testing process, as we do not have to test combinations of alleles. While a comprehensive eluted ligand dataset exists for the MHC class I peptidome, no analogous dataset exists for HLA-DRB1. Therefore, we used IEDB to gather data for alleles which were present in NetMHCIIpan-4.0’s training data, and data from a recent C1R cell line eluted ligand study for peptides binding to DRB1*12:02, an allele not represented in NetMHCIIpan-4.0’s training set (20, 21). All together, we have *n* = 45286 peptides from 10 alleles with training data in NetMHCIIpan-4.0, and *n* = 32402 peptides from allele DRB1*12:02.

**Figure 3:**
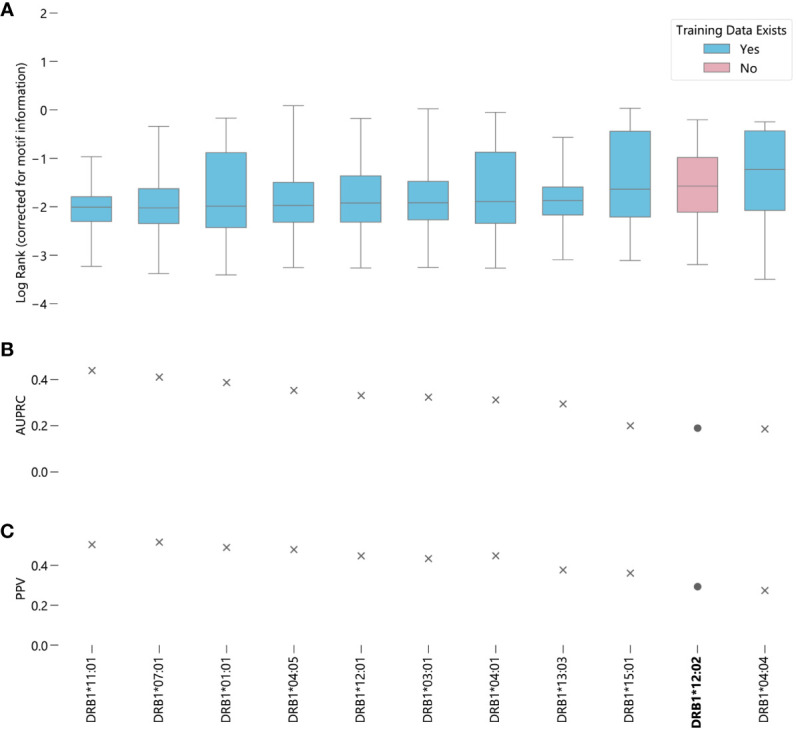
Evaluating NetMHCIIpan-4.0 performance on novel alleles: NetMHCIIpan-4.0 was tasked with separating peptides identified as true binders using LC-MS/MS (from IEDB) from randomly generated peptides for 10 HLA-DRB1 alleles with data in NetMHCIIpan-4.0’s training set, and one allele without training data. **(A)** Box-and-whisker plot of log ranks of the true peptides, corrected for entropy of the allele binding motif (lower is better). Whiskers show the middle 95% of data for each allele. Alleles with training data in NetMHCIIpan-4.0’s training dataset are shown in blue, alleles without are shown in pink. **(B)** Area under the precision-recall curve (AUPRC) for each allele. **(C)** Positive predictive value (PPV) for each allele.

In contrast to NetMHCpan-4.1, the predictions generated by NetMHCIIpan-4.0 for peptides corresponding to alleles for which it has no data are slightly worse than average, when measured by median log-rank (**Supplementary Figure S11B**). However, when measured by AUC, DRB1*12:02 ranks around average, with NetMHCIIpan-4.0 predictions for this allele better than 6 other alleles and worse than 4 other alleles (**Figure 3**) (**Supplementary Data Sheet 3**). A 95% bootstrap confidence interval for the difference in the medians between peptides corresponding to alleles with and without data in NetMHCIIpan-4.0’s training set is (-0.372, -0.321) (**Supplementary Figure S12B**). However, the middle 50% of ranks for DRB1*12:02 contains all other median ranks, suggesting the difference in prediction quality is relatively minor compared to the variability in predictions for a given allele. Furthermore, there exists an allele with data in NetMHCIIpan-4.0’s training dataset, DRB1*04:04, for which NetMHCIIpan-4.0 is less accurate at distinguishing real peptides than for DRB1*12:02. Like MHC class I predictions, in almost all cases there is a large difference between the raw EL scores between the true binders and the randomly generated peptides (**Supplementary Figure S15**), and the results hold without the correction for motif information (**Supplementary Figure S16**).

While problems of skewed datasets have affected quality of numerous other machine learning based predictions algorithms, we find no evidence this is true of NetMHCpan. By testing the ability of NetMHCpan to recall experimentally verified binding peptides to alleles for which the algorithm has no training data, we fail to conclude there exists a meaningful difference between alleles for which NetMHCpan has training data, and those for which it does not.

### 3.3 NetMHCpan training data covers a large subset of HLA allele space

As a lack of diversity in training data often leads machine learning models to overfit to their training set, we seek to understand why this does not appear to be true for NetMHCpan. Therefore, we visualize the training dataset by measuring sequence similarity between HLA alleles with frequency greater than 0.01% in any population, and use these computed similarities to perform multidimensional scaling (MDS) in order to visualize the sequence space as a two-dimensional map (25) (**Supplementary Data Sheet 4**).

**Figure 4:**
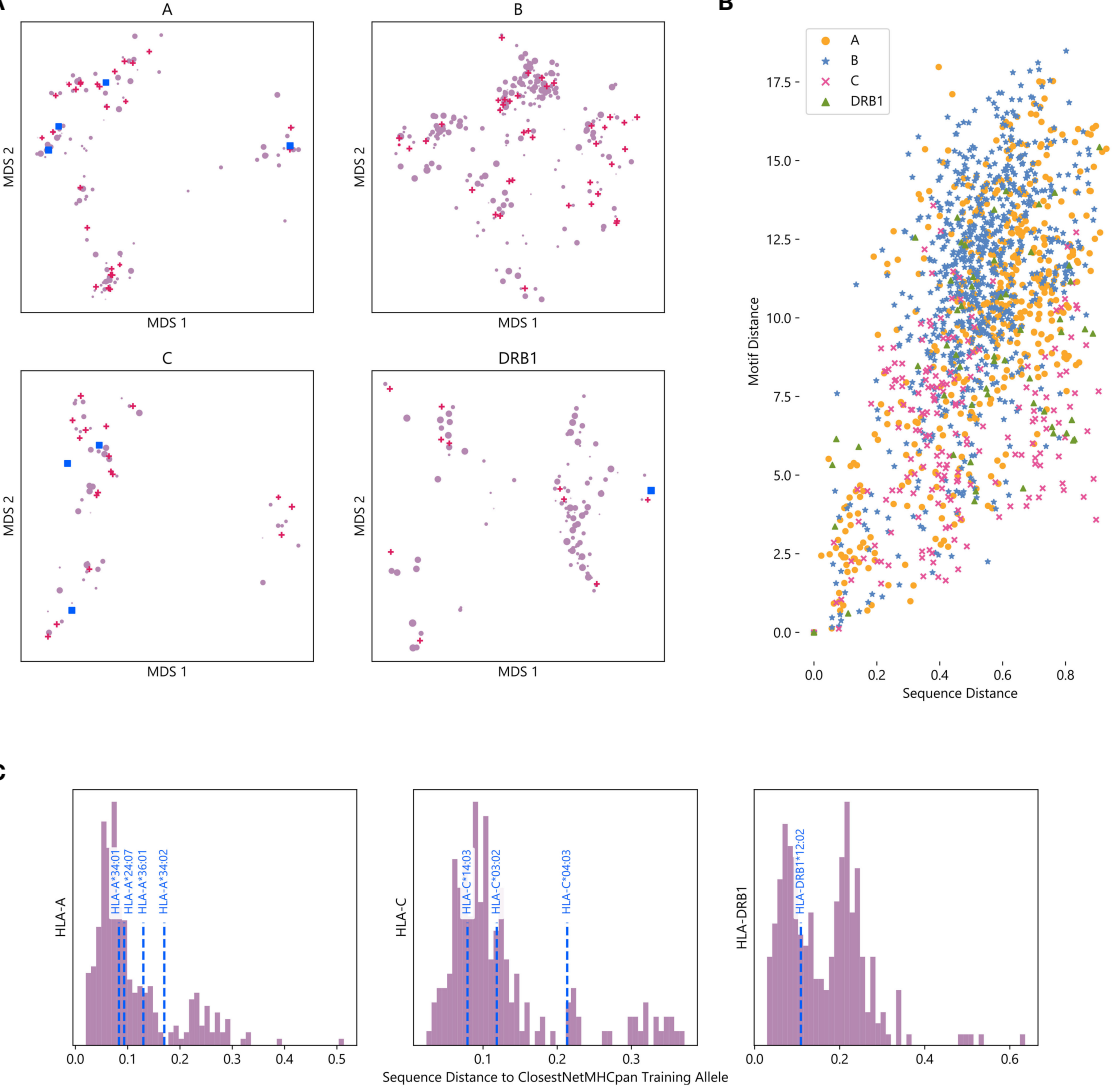
Visualizing the training space of NetMHCpan: **(A)** MDS plot of HLA alleles, with smaller distances corresponding to greater sequence similarity. Alleles included in NetMHCpan’s training data are marked with pink triangles, alleles tested in **Figures 2**, **3** with no training data are marked with blue squares, and other alleles are marked with purple circles. Marker size corresponds to maximum frequency of the allele in any NMDP population (log scale). **(B)** Comparison of sequence similarity (measured by PAM100 distance) and motif similarity (measured by symmetric KL divergence) of pairs of HLA alleles. Each point corresponds to a pair of HLA alleles for which peptide binding data exists. **(C)** Histogram of distance to closest allele to data in NetMHCpan’s training set for all alleles without training data. Alleles previously tested are shown with vertical dashed blue lines.

For all four HLA types measured, alleles tend to organize into clusters, a majority which contain at least one allele with data in NetMHCpan’s training dataset (**Figure 4A**). This suggests that while NetMHCpan may be missing data for many alleles common in non-European populations, the alleles for which it has data are sufficiently similar to the missing alleles as to allow the model to make reasonable inference about the biochemical properties of alleles without data.

Although we measure the distance between two alleles as the distance between their sequence, we recognize that measuring the distance between their associated motifs is potentially a more informative metric. As this metric is not available for alleles with no known binding peptides, we consider whether sequence distance is a good metric to approximate motif distance. For all pairs of alleles for which we have motifs, we compute the distance between their amino acid sequences and the distance between their motifs (**Figure 4B**). We find that there is moderate agreement between these two metrics (Pearson correlation coefficient *r* = 0.54), suggesting that sequence distance is a reasonable metric to use when motif distance is unavailable. We also compute an MDS using motif distance, and find that it generally agrees with our sequence MDS (**Supplementary Figure S17**).

As sequence and motif distance are correlated, measuring pairwise sequence distances between all alleles also provides context for the performance of NetMHCpan on novel alleles reported above. To measure the extent to which an allele is novel, we calculate the sequence distance to the nearest allele in the training data for each allele not in NetMHCpan’s training data (**Figure 4C**, **Supplementary Data Sheet 7**). Therefore, while the choices of which alleles without training data to test were driven by data availability, we demonstrate the alleles tested are less similar to the training data than other HLA alleles. Thus, the accuracy of NetMHCpan’s predictions for these alleles is not driven by greater than average similarity of these alleles to alleles found in the training dataset. While we lack a sufficient number of alleles to establish a relationship, we hypothesize that sequence and motif distance between an allele with no training data and the nearest allele is the training set are negatively correlated with performance (**Supplementary Figure S18**).

### 3.4 NetMHCpan correctly identifies MHC residues involved in peptide binding

Finally, we aim to understand the extent to which NetMHCpan identifies residues structurally involved in peptide binding. As NetMHCpan allows for direct input of an MHC protein sequence, we perform an experiment in which we mutate each residue of a given HLA sequence individually, and measure how much NetMHCpan’s EL scores for experimentally verified peptides change compared to the unmodified sequence (**Supplementary Data Sheet 5**). We focus on three case studies, HLA-A*34:01, HLA-C*04:03, and HLA-DRB1*12:02, as these alleles constitute the worst-performing allele for each HLA type.

**Figure 5:**
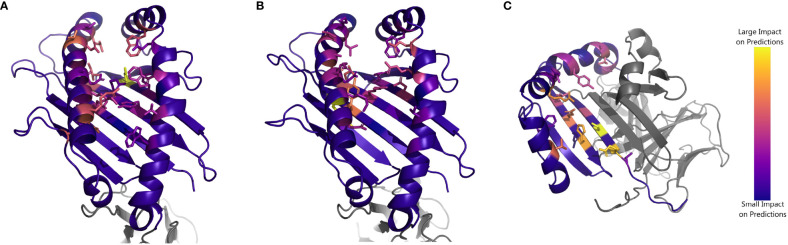
Impact of substituting residues on NetMHCpan predictions for HLA alleles of interest: Structure of **(A)** HLA-A*34:01 **(B)** HLA-C*04:03 and **(C)** HLA-DRB1*12:02. Residues are colored by impact of substitution on NetMHCpan predictions. Yellow resides indicate a large change to NetMHCpan predictions when replaced, purple resides indicate a small change. Sidechains are shown for residues of interest.

In each case, the MHC residues which have the greatest impact on NetMHCpan’s prediction are all residues that make physical contact with the peptide (**Figure 5**, **Supplementary Tables S6**-**S8**). This suggests that the accuracy of NetMHCpan’s predictions on novel alleles is partly driven by its ability to selectively pay attention to residues involved with the physical process of binding. Of special interest is the observation that many residues which affect the predictions for peptides binding to DRB1*12:02 are residues previously identified to determine the binding motif of DR12, namely, 13G, 57V, 70D, 71R, 74A, and 86V (21). Therefore, we conclude NetMHCpan implicitly learns the MHC residues structurally involved in binding, and its ability to generalize these findings to novel alleles increases its prediction accuracy.

## 4 Discussion

We report NetMHCpan fails to include a geographically diverse set of HLA alleles in its training data. We find individuals from underrepresented populations, predominantly from Asia, are twenty times more likely to carry HLA alleles not present in NetMHCpan’s training data. Furthermore, we observe correlation between population representation between all four alleles measured, suggesting that the dataset bias is a result of systemic underrepresentation of minority groups in the NetMHCpan training dataset.

Numerous previous examples of training dataset racial bias affecting machine learning model predictions led us to hypothesize NetMHCpan would make less accurate predictions on alleles which were not present in its training dataset (12–14). Furthermore, previous work showed NetMHCpan is subject to systemic biases regarding hydrophobicity, suggesting that other biases may be lurking (11). Unexpectedly, we fail to find evidence that there is a substantial difference in the ability of NetMHCpan to discriminate experimentally verified binding peptides from randomly generated peptides. Instead, we observe a slight increase in the prediction ability for MHC class I alleles with no data present in the training set, and only a slight decrease for MHC class II alleles. While both effects are statistically significant, we allege neither is large enough to have a substantial effect on prediction quality.

To explain this unexpected result, we characterize the sequence space of common HLA alleles. While NetMHCpan’s training dataset fails to include many alleles common in underrepresented populations, we show that the alleles for which training data exist are well-distributed throughout sequence space. We thus hypothesize that MHC sequence diversity in the training dataset partially explains the failure to observe a drop in prediction quality. Furthermore, we establish a connection between the residues that impact NetMHCpan’s predictions and the residues that physically contact the peptide for three HLA alleles not present in NetMHCpan’s training data.

The discrepancies in the diversity of HLA eluted ligand datasets that compelled this study also constitute a major limitation, as only eight novel HLA alleles were tested, with no novel HLA-B alleles. Furthermore, our study design was limited to only testing one allele at a time, and so we did not investigate complex effects that could be associated with linkage disequilibrium in MHC class II molecules formed by two interacting chains, including HLA-DQ and HLA-DP (29). We only tested the ability of NetMHCpan to distinguish experimentally verified peptides from randomly generated peptides, and did not perform any experiments to characterize the model’s ability to predict binding affinity. Finally, NetMHCpan is closed source, and so we were unable to view the internal network structure, needing to rely on an occlusion sensitivity-like metric to determine how the network makes predictions.

We present evidence of a strong bias in NetMHCpan’s training dataset toward European Caucasian individuals. While we fail to find evidence this bias affects the accuracy of NetMHCpan’s predictions, the bias in the training dataset highlights the need for MHC eluted ligand datasets that contain data for alleles for underrepresented populations. Furthermore, given the outsized impact of NetMHCpan on the training data generated for other MHC binding prediction tools, future work must investigate the composition of training datasets and potential bias in other tools (30). Finally, we recommend all tools that utilize a dataset involving HLA alleles as part of their pipeline clearly report the composition of any datasets they utilize for training, and perform additional testing in the presence of biased training data to ensure model predictions do not substantially decline for underrepresented groups.

## Data availability statement

Publicly available datasets were analyzed in this study. This data can be found here: https://frequency.nmdp.org/; https://services.healthtech.dtu.dk/suppl/immunology/NAR_NetMHCpan_NetMHCIIpan/; https://services.healthtech.dtu.dk/suppl/immunology/NetMHCpan-4.0/; https://www.ebi.ac.uk/ipd/imgt/hla/; ftp://massive.ucsd.edu/MSV000084172/.

## Ethics statement

Ethical approval was not required for the study involving humans in accordance with the local legislation and institutional requirements. Written informed consent to participate in this study was not required from the participants or the participants’ legal guardians/next of kin in accordance with the national legislation and the institutional requirements.

## Author contributions

TA: Conceptualization, Data curation, Investigation, Methodology, Visualization, Writing – original draft, Writing – review & editing. AS: Writing – review & editing. GV: Supervision, Writing – review & editing. JC: Supervision, Writing – review & editing. MR: Funding acquisition, Project administration, Supervision, Writing – review & editing.

## Supplementary material

Supplementary Data Sheet 1 Supplementary Figures and Tables.

Supplementary Data Sheet 2 Raw data for **Figure 1**.

Supplementary Data Sheet 3 Raw data for **Figures 2** and **3**.

Supplementary Data Sheet 4 Raw data for **Figure 4**.

Supplementary Data Sheet 5 Raw data for **Figure 5**.

Supplementary Data Sheet 6 Results of Gibbs Clustering.

Supplementary Data Sheet 7 Distance matrix between HLA sequences.
