# Supplementary material for: Evaluating NetMHCpan performance on non-European HLA alleles not present in training data
Source: Front Immunol. 2024 Jan 16;14:1288105. doi: 10.3389/fimmu.2023.1288105 (PMC10825027; doi:10.3389/fimmu.2023.1288105)
Supplement: Supplementary Data Sheet 1 — Supplementary Figures and Tables. [file DataSheet_1.pdf]

Supplementary Material

SUPPLEMENTARY TABLES AND FIGURES

Tables

Allele	Peptides	Unique Cores
A*01:01	1175	1079
A*02:01	3266	3108
A*02:02	3438	3249
A*02:03	2763	2618
A*02:04	2147	2001
A*02:05	3335	3137
A*02:06	2365	2291
A*02:07	3849	3572
A*02:11	2248	2060
A*03:01	1818	1703
A*11:01	4350	4080
A*11:02	2759	2628
A*23:01	2740	2570
A*24:02	2353	2244
A*24:07	1440	1366
A*25:01	1095	1045
A*26:01	1486	1336
A*29:02	1074	1021
A*30:01	1379	1326
A*30:02	2463	2297
A*31:01	1084	1043
A*32:01	2162	2026
A*33:01	2392	2259
A*33:03	2949	2741
A*34:01	2343	2208
A*34:02	3539	3345
A*36:01	2001	1835
A*66:01	2075	2007
A*68:01	1861	1763
A*68:02	1787	1683
A*74:01	2527	2393

Table S1. Number of experimentally verified binding peptides in the testing set for each HLA-A allele. The unique cores column corresponds to the number of unique binding cores prediction by NetMHCpan.

Allele	Peptides	Unique Cores
C*01:02	1497	1434
C*02:02	1119	1059
C*03:02	1304	1230
C*03:03	3030	2820
C*03:04	2495	2363
C*04:01	2115	1901
C*04:03	1149	1085
C*05:01	1549	1494
C*06:02	1692	1605
C*07:01	931	876
C*07:02	1205	1142
C*07:04	918	813
C*08:01	2007	1851
C*08:02	3428	3199
C*12:02	1576	1468
C*12:03	2356	2188
C*14:02	1532	1390
C*14:03	3116	2846
C*15:02	3608	3349
C*16:01	3370	3123
C*17:01	1075	1043

Table S2. Number of experimentally verified binding peptides in the testing set for each HLA-C allele. The unique cores column corresponds to the number of unique binding cores prediction by NetMHCpan.

Allele	Peptides	Unique Cores
DRB1*01:01	15013	4873
DRB1*03:01	4664	1431
DRB1*04:01	9200	2872
DRB1*04:04	2421	1176
DRB1*04:05	5147	1415
DRB1*07:01	5217	1867
DRB1*11:01	3683	1079
DRB1*12:01	2660	1048
DRB1*12:02	32458	7648
DRB1*13:03	3322	971
DRB1*15:01	5253	2003

Table S3. Number of experimentally verified binding peptides in the testing set for each HLA-DRB1 allele. The unique cores column corresponds to the number of unique binding cores prediction by NetMHCpan.

Code	Description	Group
AAFA	African American	AFA
AFB	African	AFA
AINDI	South Asian Indian	API
AISC	American Indian — South or Central Am.	NAM
ALANAM	Alaska native or Aleut	NAM
AMIND	North American Indian	NAM
CARB	Caribbean black	AFA
CARHIS	Caribbean hispanic	HIS
CARIBI	Caribbean Indian	NAM
EURCAU	European caucasian	CAU
FILII	Filipino	API
HAWI	Hawaiian or other Pacific Islander	API
JAPI	Japanese	API
KORI	Korean	API
MENAFC	Middle Eastern or N. Coast of Africa	CAU
MSWHIS	Mexican or Chicano	HIS
NCHI	Chinese	API
SCAHIS	Hispanic — South or Central American	HIS
SCAMB	Black — South or Central American	AFA
SCSEAI	Southeast Asian	API
VIET	Vietnamese	API

Table S4. Full descriptions for codes given by NMDP project

	A	B	C	DRB1
A	-	0.750	0.401	0.238
B		-	0.684	0.732
C			-	0.643
DRB1				-

Table S5. Correlations between absence of HLA alleles in population groups

Pos	AA	Change
33	Y	0.157
48	A	0.039
69	M	0.230
86	R	0.218
87	N	0.260
90	K	0.155
91	V	0.109
93	A	0.080
94	Q	0.107
97	T	0.064
98	D	0.153
100	V	0.262
101	D	0.194
104	T	0.107
105	L	0.219
119	I	0.215
121	R	0.063
123	Y	0.123
138	Q	0.113
140	D	0.420
167	T	0.025
171	W	0.210
176	E	0.083
180	W	0.137
182	A	0.034
187	T	0.123
191	W	0.072

Table S6. Average change in NetMHCpan-4.1 EL score for experimentally binding peptides under residue substitution for peptides binding to HLA-A*34:01

Pos	AA	Change
33	Y	0.182
48	A	0.032
86	R	0.017
87	E	0.097
90	K	0.075
91	Y	0.281
93	R	0.093
94	Q	0.064
97	A	0.095
98	D	0.159
100	V	0.112
101	N	0.045
104	K	0.068
105	L	0.099
119	L	0.155
121	R	0.112
123	F	0.032
138	N	0.117
140	F	0.081
171	W	0.071
176	E	0.143
180	R	0.122
182	A	0.035
187	T	0.050
191	W	0.043

Table S7. Average change in NetMHCpan-4.1 EL score for experimentally binding peptides under residue substitution for peptides binding to HLA-C*04:03

Pos	AA	Change
8	L	0.045
9	E	0.190
11	S	0.216
13	G	0.103
26	L	0.099
28	E	0.143
30	H	0.173
47	F	0.037
57	V	0.135
70	D	0.042
71	R	0.161
74	A	0.065
77	T	0.066
78	Y	0.088
85	A	0.083
86	V	0.110
89	F	0.011
90	T	0.082
89	T	0.082

Table S8. Average change in NetMHCIIpan-4.0 EL score for experimentally binding peptides under residue substitution for peptides binding to HLA-DRB1*12:02

Figures

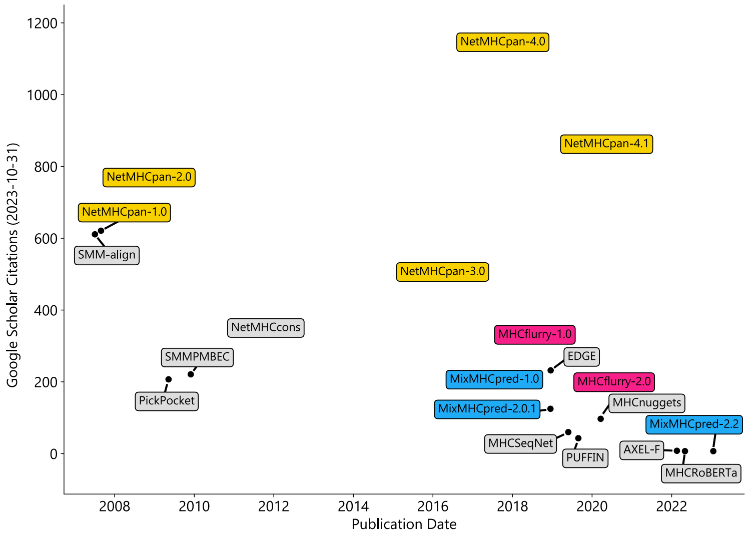

Figure S1. Google Scholar citation count (accessed Oct. 31, 2023) for MHC-peptide binding predictors versus publication date. Tools with multiple versions are shown in the same colors, tools without multiple versions are shown in gray.

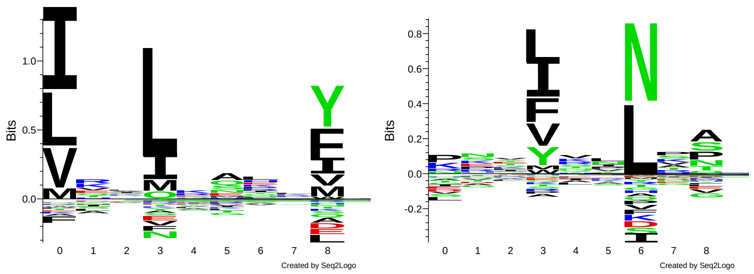

Figure S2. Sequence logos for HLA-DR cores after Gibbs clustering. The left group corresponds to HLA-DRB1*12:02, the right to HLA-DRB3*02:02.

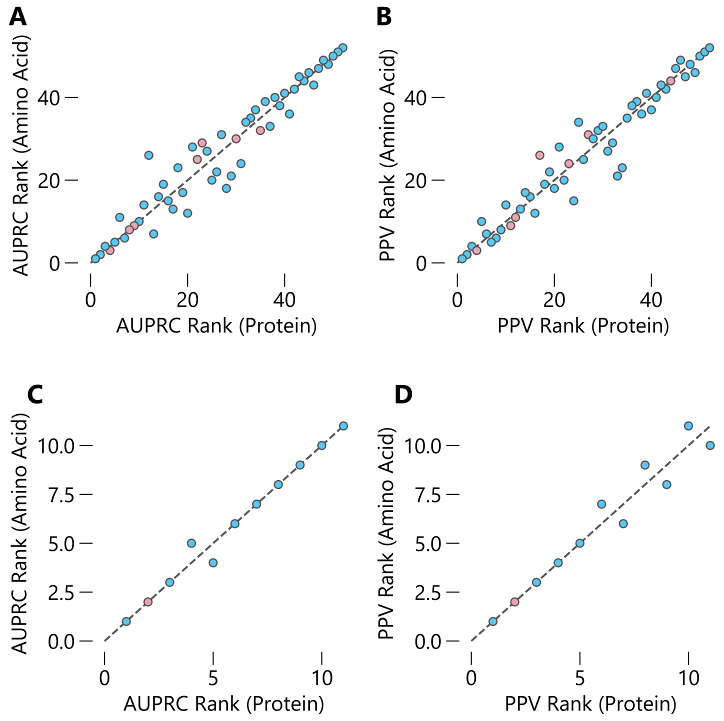

Figure S3. Relative ranks of AUPRC and PPV for each allele for (A,B) NetMHCpan-4.1 and (C, D) NetMHCIIpan-4.0 for different negative control peptide sampling methods. On the x-axis is the rank of the allele when sampling entire peptides from human proteins, on the y-axis is the rank of the allele when sampling random strings of amino acids (with frequencies equal to that of the human proteome). Blue dots are alleles with training data, pink dots are alleles without training data.

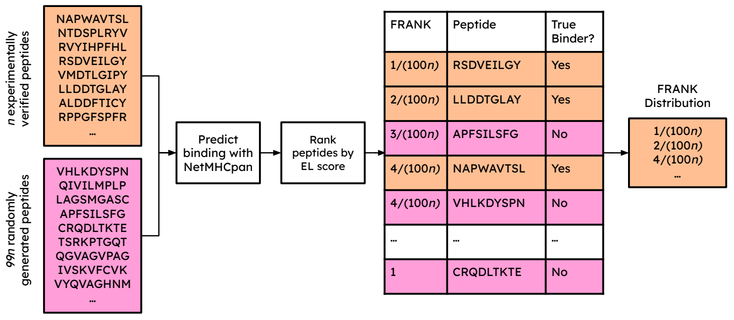

Figure S4. Visual overview of the NetMHCpan evaluation process. We start with a dataset of n peptides experimentally verified to bind to a given HLA allele. Next, we randomly generate $99n$ peptides to serve as a negative control. We ask NetMHCpan to predict binding for all peptides, and then calculate the fractional rank (FRANK) of the experimentally verified peptides. We measure the quality of the predictions by the distribution of the FRANKs for experimentally verified peptides, with lower FRANKs meaning better predictions.

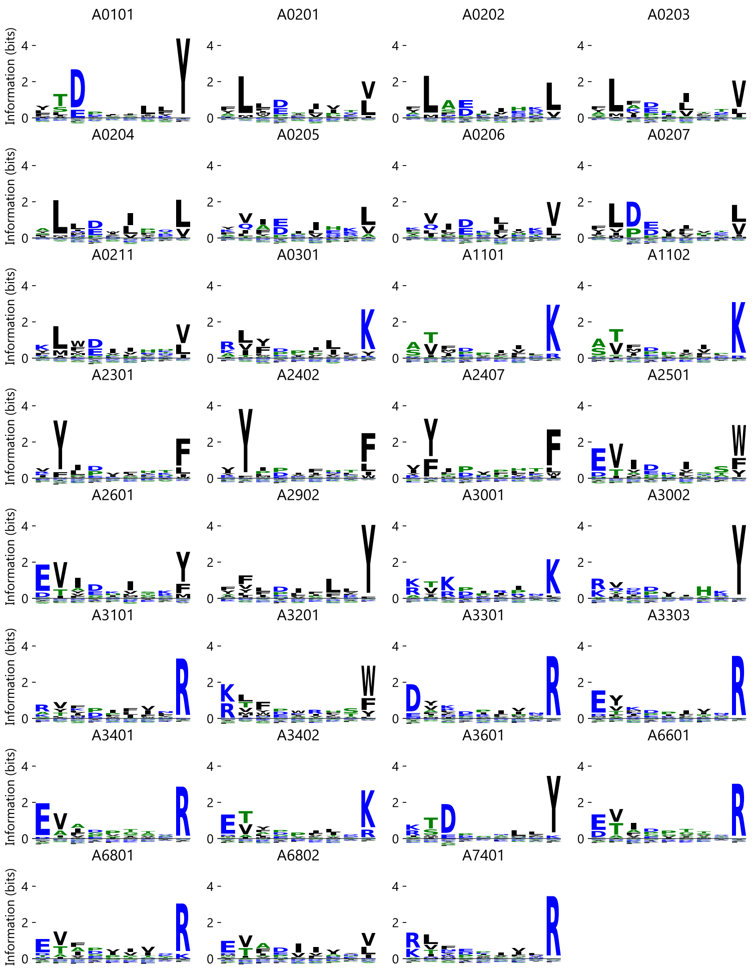

Figure S5. Sequence motifs for each HLA-A allele evaluated in our study. Height is proportional to KL divergence between frequency of amino acids at the given position in known binding peptides and human proteome amino acid frequencies.

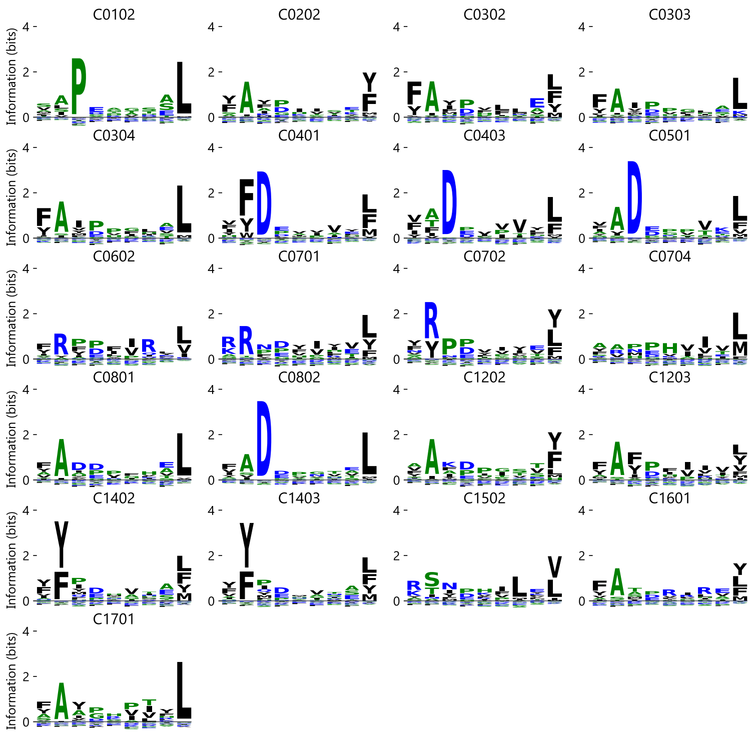

Figure S6. Sequence motifs for each HLA-C allele evaluated in our study. Height is proportional to KL divergence between frequency of amino acids at the given position in known binding peptides and human proteome amino acid frequencies.

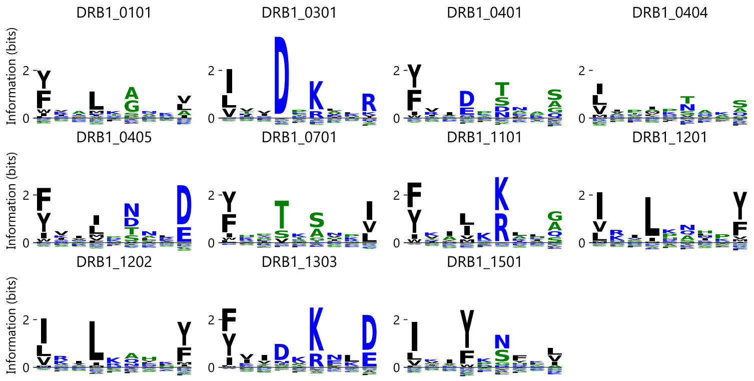

Figure S7. Sequence motifs for each HLA-DRB1 allele evaluated in our study. Height is proportional to KL divergence between frequency of amino acids at the given position in known binding peptides and human proteome amino acid frequencies.

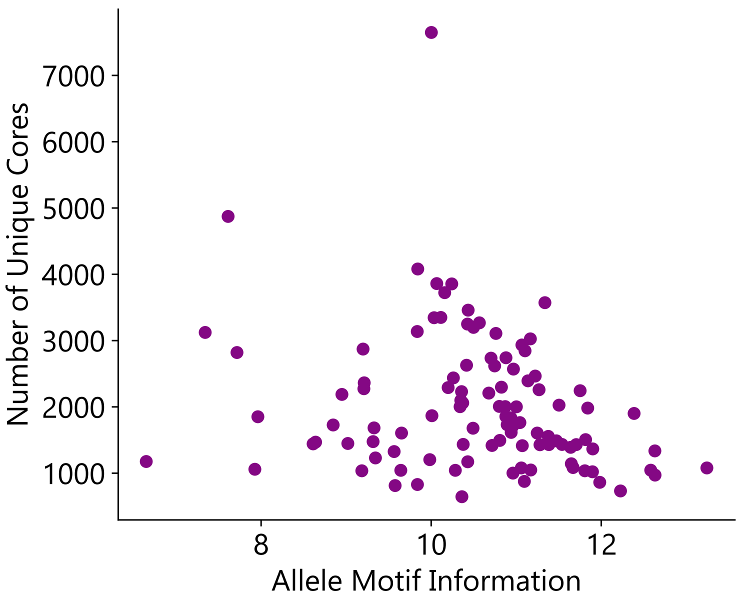

Figure S8. Number of unique peptide cores versus KL motif information for every allele tested.

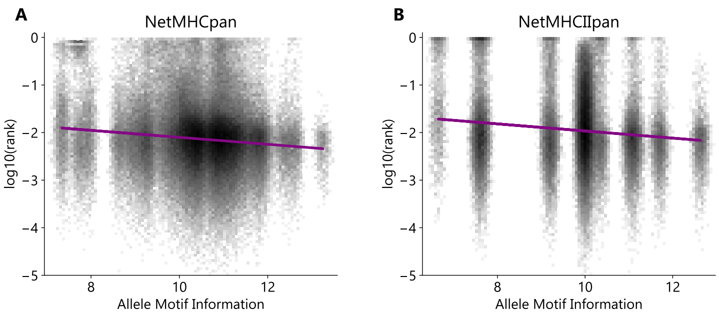

Figure S9. Two-dimensional histogram of (A) NetMHCpan-4.1 and (B) NetMHCIIpan-4.0 rank predictions vs. allele motif information. Fractional rank of the peptides is plotted against the motif information of the associated allele. Jittering is performed on the x-axis to allow points to be more easily distinguished, and colors are log-transformed.

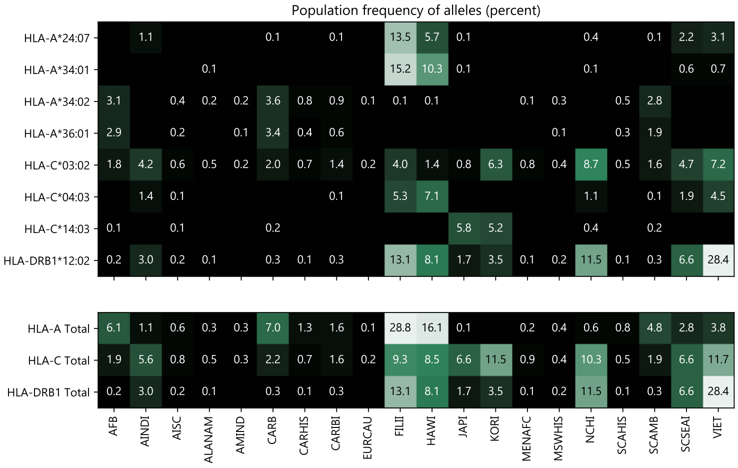

Figure S10. NMDP population frequencies of the alleles from Sarkizova et. al. that are not present in the NetMHCpan training dataset, per 100. Values of less than 0.1% are omitted.

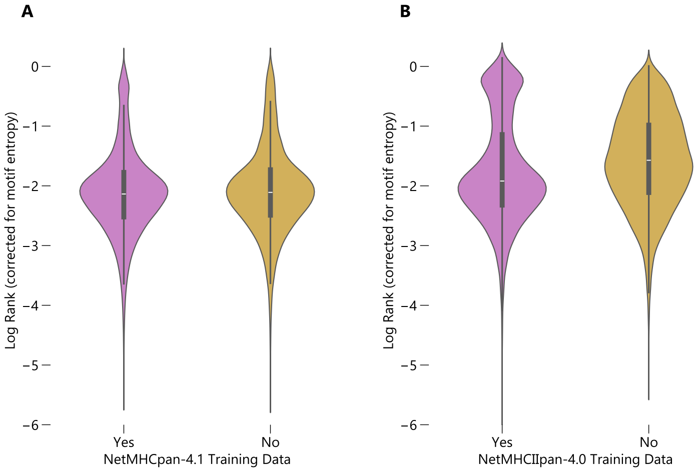

Figure S11. Distribution of entropy-corrected log ranks for peptides corresponding to HLA alleles with and without data in (A) NetMHCpan-4.1's and (B) NetMHCIIpan-4.0's training set.

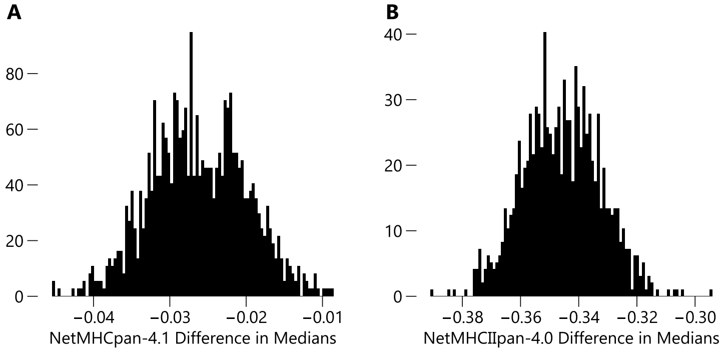

Figure S12. Bootstrap distributions of the median of log ranks of predictions made with training data minus the median of log ranks of predictions made without training data. Positive values indicate the model performs better without training data. (A) Distribution for NetMHCpan-4.1. (B) Distribution for NetMHCIIpan-4.0.

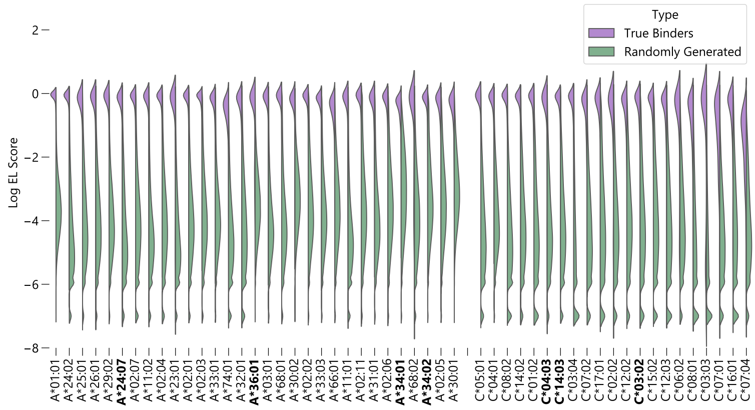

Figure S13. Distributions of the log of NetMHCpan-4.1's EL score. Purple indicates the peptides that are experimentally verified to bind to the given allele, green indicates randomly generated peptides. Bolded alleles are alleles for which NetMHCpan-4.1 had no training data.

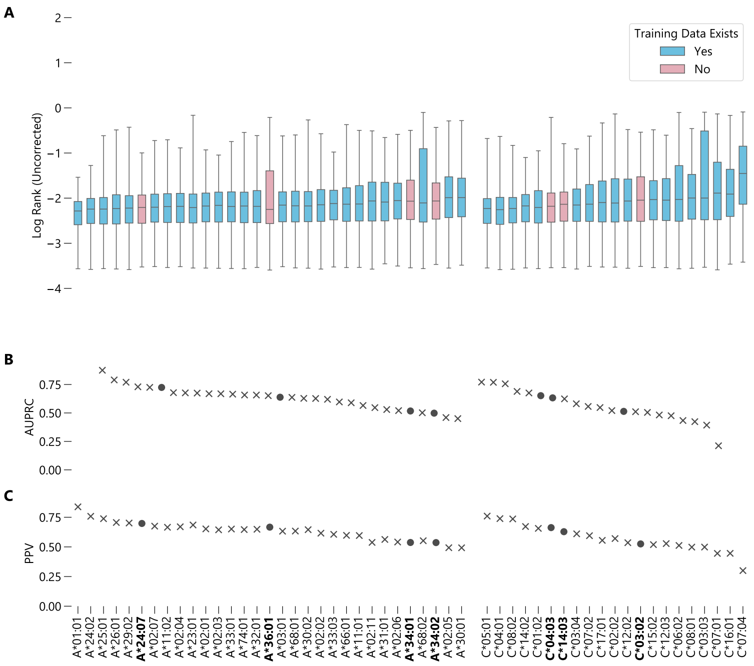

Figure S14. Performance of NetMHCpan-4.1 on tested alleles without a correction for motif information. (A) Box-and-whisker plot of log ranks of the true peptides, corrected for entropy of the allele binding motif (lower is better). Whiskers show the middle 95% of data for each allele. Alleles with training data in NetMHCpan-4.1's training dataset are shown in blue, alleles without are shown in pink. (B) Area under the precision-recall curve (AUPRC) for each allele.

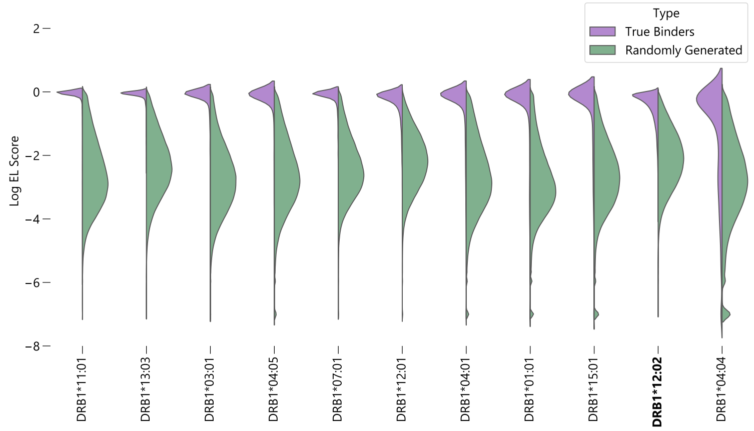

Figure S15. Distributions of the log of NetMHCIIpan-4.0's EL score. Purple indicates the peptides that are experimentally verified to bind to the given allele, green indicates randomly generated peptides. Bolded alleles are alleles for which NetMHCIIpan-4.0 had no training data.

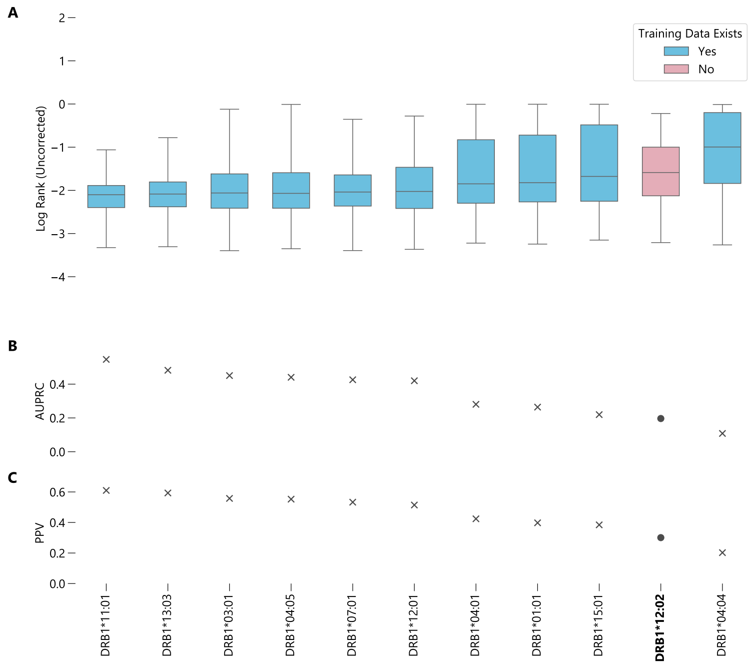

Figure S16. Performance of NetMHCIIpan-4.0 on tested alleles without a correction for motif information. (A) Box-and-whisker plot of log ranks of the true peptides, corrected for entropy of the allele binding motif (lower is better). Whiskers show the middle 95% of data for each allele. Alleles with training data in NetMHCIIpan-4.0's training dataset are shown in blue, alleles without are shown in pink. (B) Area under the precision-recall curve (AUPRC) for each allele.

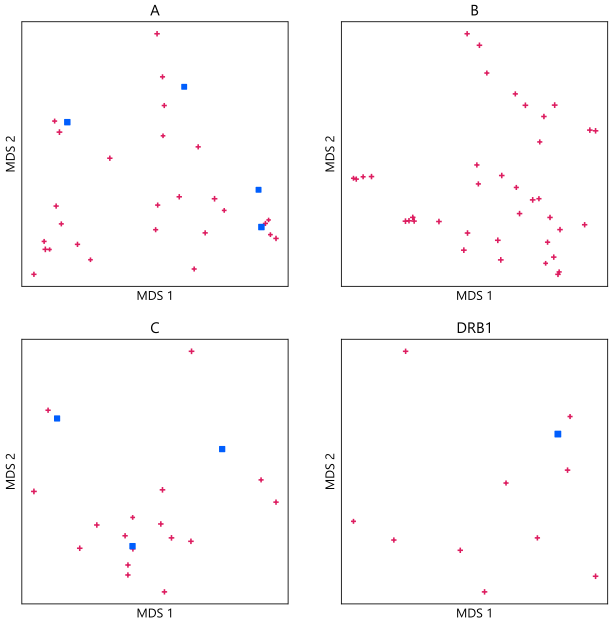

Figure S17. MDS performed on motif distance for alleles which have associated binding peptides. Alleles tested in Figures 2 and 3 with no training data are marked with blue squares, and other alleles are marked with pink crosses.

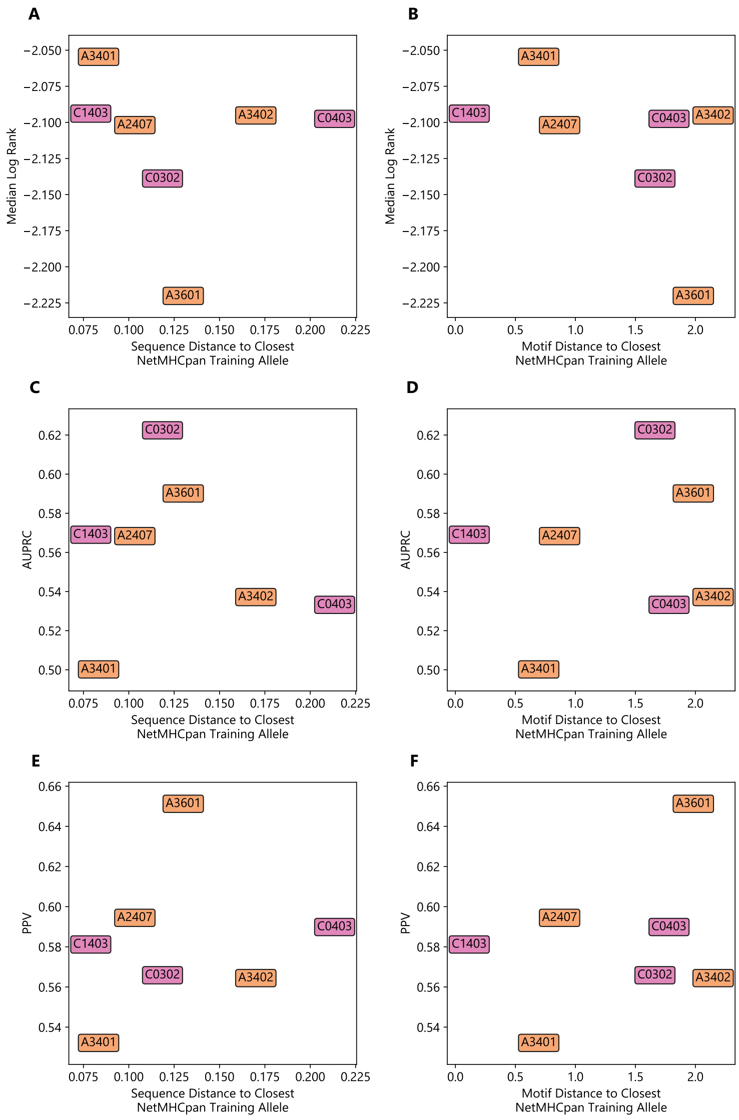

Figure S18. NetMHCpan median log rank (A,B), AUPRC (C,D), and PPV (E,F) versus minimum sequence distance and KL motif distance to the closest allele in the NetMHCpan training dataset for $n = 7$ HLA class I alleles without data in NetMHCpan-4.1's training set. Lower median log rank is better, higher AUPRC and PPV is better. Color corresponds to HLA type.
